# The Different Roads to Maintain Telomeres in Cancer Cells

**DOI:** 10.3390/genes11121525

**Published:** 2020-12-21

**Authors:** Rosario Perona

**Affiliations:** Instituto de Investigaciones Biomédicas CSIC/UAM, C/Arturo Duperier, 4 Madrid, Centro de Investigación Biomédica en Red (CIBER) de Enfermedades Raras, 28029 Madrid, Spain; rperona@iib.uam.es

Telomeres are the protective structures at the ends of linear chromosomes that progressively shorten each time that a cell divides, which is in part caused by the end-replication problem [1]. In the absence of a mechanism to maintain telomere length, telomeres in cells would be progressively shortened, leading to a phenomenon known as replicative senescence.

Different diseases deficient in telomerase activity, defined as telomeropathies, induced by mutations in genes of the telomerase or shelterin complexes, are characterized by premature senescence in stem cells, giving rise to a premature aging of different tissues. On the opposite side of these diseases is cancer, since cancer cells are immortalised. Therefore, the question in cancer is how do cancer cells maintain telomeres? The general answer is that cancer cells develop different mechanisms to upregulate telomerase activity or alternative methods to maintain telomere length.

In the article “Telomere Maintenance Mechanisms in Cancer” Bordeira T. and collaborators [2], in addition to providing a comprehensive review about general mechanisms of telomere maintenance and their relevance, also contributed with an exhaustive review of the different mechanisms used by cancer cells to induce telomerase activity. The activation of these mechanisms is necessary for any cell to continue dividing and progressing to a malignant phenotype.

Genetic instability and DNA damage are necessary events in carcinogenesis that activate signalling pathways that may lead to amplification and/or increased expression of telomerase, allowing tumour cells to survive cellular crisis and achieve immortality, one of the major hallmarks of cancer. Therefore, there are several mechanisms of telomere length maintenance in tumour cells.

TERT promoter mutations (TERTpm) were the first mechanism described, and are the most frequent somatic non-coding alterations harboured by a wide spectrum of human tumours, among them CNS, thyroid, skin, bladder, and liver. The most frequent mutations in TERTp creates an 11 bp nucleotide stretch that contains a consensus binding site for E-twenty-six (ETS) transcription factors within the promoter region. This is consistent with the relevance of these mutations in tumours with dysregulation of mitogen-activated protein kinase (MAP kinase) signalling such as melanoma, thyroid, and skin. In the case of cutaneous melanoma, this alteration associates with BRAF-mutations and with poorer prognostic features. In most tumour types, TERTpm appear at the last steps of cancer progression and are associated with worst prognosis and poor patient survival. In those tumours with high mutational rates such as hepatocellular carcinoma (HCC), the potential involvement of TERTp mutations in the malignant transformation of HC adenocarcinomas has been suggested. The same role of TERTp mutations was found in basal cell carcinoma and invasive squamous cell carcinoma (SCC).

Increased TERT and TERC gene copy number has been associated with increased expression of both genes in the absence of TERTp mutations. Remarkably, an increase in gene copy number correlates with tumour progression, as TERT amplifications were detected only in invasive melanomas. In some tumours TERTp mutations and TERT gene amplifications are mutually exclusive. Such is the case of HCC, where 15% of these tumours showed TERT amplifications. Amplifications of the TERC gene have been detected in oesophageal carcinoma, lung SCC, and ovarian and cervical tumours.

Genome-wide association studies (GWAS) have strongly contributed to the identification of common variants in TERT locus. Several single nucleotide polymorphisms (SNPs) in this region have been consistently associated with increased risk for developing various types of tumours. These SNPs may arise either in intronic or exonic sequences of TERT or the promoter region.

TERT gene rearrangements can result in tandem duplications, inversions, interchromosomal changes, amplification, and deletions. This is the case of neuroblastoma defining a subgroup of high-risk tumours with a particularly poor outcome. The degree at which the TERT promoter is methylated also plays a role in carcinogenesis. Hypermethylated states can prevent binding of transcriptional repressors. Overall, the better characterized TERTp hypermethylation at specific CpG islands, also named as THOR (TERT hypermethylated oncological region), has been reported to have diagnostic and prognostic value in prostate and pancreatic (exocrine and endocrine) cancers. Methylation of the TERTp has also been suggested as a biomarker for malignancy and patient outcome in paediatric gliomas, such as pilocytic astrocytoma, medulloblastoma, ependymoma, and choroid plexus carcinoma, among others.

Alternative lengthening of telomeres (ALT) is a telomerase-independent mechanism of telomere lengthening. ALT-positive cells are dependent on the activation of a homologous recombination DNA-repair mechanism to maintain telomere length. Tumours of mesenchymal origin are reported to activate ALT most frequently, which may be explained by the fact that mesenchymal stem cells express minimal or undetectable telomerase activity.

Finally, these authors emphasized some intriguing questions that remain to be answered, such as the reasons behind the gradual increase in telomere repair activation with grade progression; the high-grade dependence of some histological types for specific telomere repair activation; the homogenous distribution of telomere repair activation frequencies among very different tumour grades and the apparent absence of telomere repair activation in some tumours. These unsolved questions should be a matter of future studies and might get to the discovery of new mechanisms for telomere repair that eventually may contribute to tumour staging and treatment opportunities.
